# Supplementary material for: Deep fiber clustering: Anatomically informed fiber clustering with self-supervised deep learning for fast and effective tractography parcellation
Source: Neuroimage. Author manuscript; Available in PMC 2024 Mar 22. (PMC10958986; doi:10.1016/j.neuroimage.2023.120086)
Supplement: supplementary material [file NIHMS1972403-supplement-supplementary_material.pdf]

Supplementary Material 1

We conducted experiments to investigate the clustering performance given different numbers of sampled points ($n_p \in [5, 35]$) per fiber. For each selected number of points, a DGCNN model was trained and tested on the 50 HCP testing subjects. The performance was evaluated with the four cluster quality metrics (DB index, WMPG, TAPC and TSPC), an efficiency metric (execution time) and two memory metrics (computer and GPU memory usages). As shown in Fig. S1, the four evaluation metrics of cluster quality are similar across different tested values of n_p, probably due to the fact that a smaller number of points includes enough information for fiber clustering. However, the prediction time and memory usage increased obviously with increased n_p, especially for large n_p. In this study, we set n_p as 14 because this number enables good performance with relatively low computational costs in terms of inference time and memory usage.

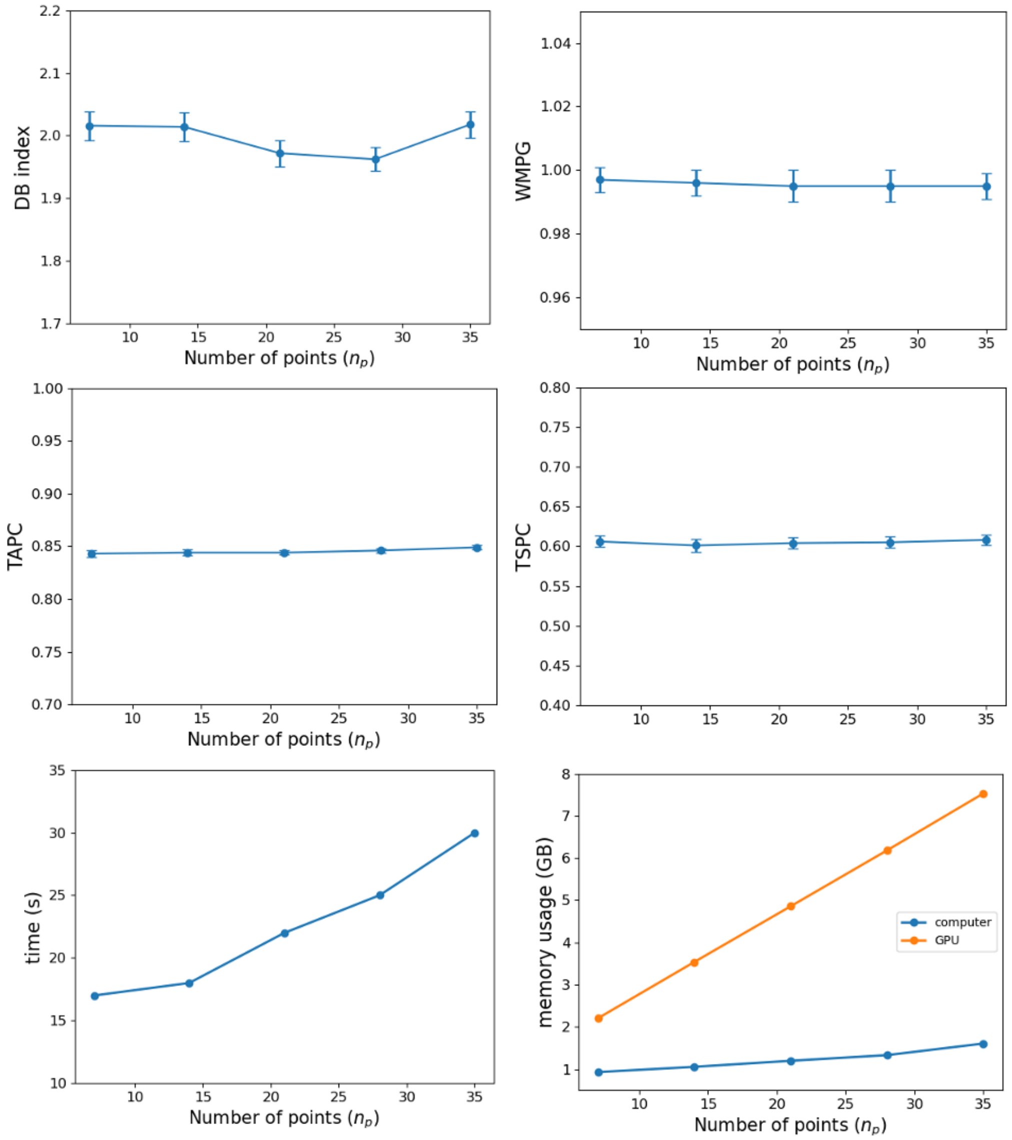

Fig. S1: DFC performance with increasing number of points along a fiber. Performance is evaluated with DB index, WMPG, TAPC, TSPC, execution time and memory usages.
